# Maternal Economic Well-Being and Mental Health among Young Adult Children: Race/Ethnicity

**DOI:** 10.3390/ijerph18115691

**Published:** 2021-05-26

**Authors:** Jaewon Lee

**Affiliations:** Department of Social Welfare, Inha University, Incheon 22212, Korea; j343@inha.ac.kr

**Keywords:** maternal economic well-being, mental health, young adult children, racial/ethnic disparities

## Abstract

This study aimed to examine the relationship between maternal economic well-being and children’s mental health outcomes in adulthood and to consider the moderating effect of race/ethnicity. This study used data from the National Longitudinal Survey of Youth 1979 and the National Longitudinal Survey of Youth 79 for Children and Young Adults. The two datasets were merged, and 4224 pairs were selected for the final sample. Ordinary linear regression and logistic regression analyses were used. Poverty and lower net worth among mothers were positively associated with their children’s depression in young adulthood. Race/ethnicity moderated the relationship between maternal poverty and children’s depression. Therefore, women’s economic resources may be an important factor in the development of mental health issues among their children in young adulthood. Developing anti-poverty policies that target women may assist in reducing depressive symptoms in their children once they reach young adulthood, specifically for non-Hispanic White children.

## 1. Introduction

Mental health in early adulthood is influenced by a variety of factors, such as economic status, marriage, and gender [1,2,3]. The cohesion and strength of the relationship between mothers and children influences the psychological health of the children in young adulthood. For instance, children whose mothers have experienced depression are at increased risk for depression when they enter adulthood [4]. However, maternal economic well-being has not yet been considered as an indicator influencing depression in young adults, even though the number of females in the labor market has increased over time [5]. In addition, while there is a large amount of research on the racial and ethnic differences in mental health [6,7], few studies have focused on racial and ethnic disparities in the relationship between maternal economic well-being and the mental health of their children in young adulthood.

Economic well-being can be defined in a number of ways, such as financial stability, the ability to maintain one’s lifestyle, or satisfaction with one’s economic assets [8,9]. In this study, economic well-being is defined through levels of economic hardships based on assets and poverty status. As economic challenges increase stressors and make it more difficult to meet one’s basic needs [10], young adults with mothers who have poor economic well-being may struggle due to a lack of financial support from their mothers, which may also contribute to mental health problems. In other words, their mental health may be influenced by a dearth of maternal financial support and economic resources [11]. Moreover, previous research has examined the relationship between mental health and economic well-being, and researchers identified racial and ethnic disparities in both economic well-being and mental health status [12,13]. However, few studies have examined the effect of race/ethnicity on this relationship. More specifically, little is known about the effects that intergenerational transmission, particularly between mother and child, would have on the relationship described, and if that too would show racial/ethnic differences. Therefore, this study aims to examine the relationship between maternal economic well-being and young adult children’s mental health and to investigate whether this association differs by race/ethnicity.

### 1.1. Women’s Economic Well-Being

Economic well-being is a critical factor in maintaining daily life because individuals with low economic well-being due to poverty or unemployment might feel uncertainty because of their situation and experience a sense of losing control [14]. Over time, rates of women’s labor force participation have increased rapidly [5,15]. In addition, as gender equality in the labor market continues to increase [16] and women’s economic activities increase, barriers to women’s employment and income disparities between men and women are decreasing [17,18]. Women have improved their qualifications for jobs and are employed in higher-level positions [19,20]. Women’s increasing labor market participation contributes to earning a livable wage and their households’ purchasing power [21]. As such, because women’s economic and workforce participation have become increasingly important to families’ prosperity, women’s economic well-being should be considered in depth.

Among the factors affecting economic well-being, having more assets without debt is a positive influence [22]. Individuals with a positive net worth tend to have financial security, leading them to feel safer if they are suddenly faced with job displacement or an interruption of income [23]. Poverty is another indicator of economic well-being [24]. Individuals living in poverty are more likely to have financial stressors in their daily lives as well as a lower quality of life than those who are not in poverty. Given that African Americans and Hispanics are more likely to be in poverty compared to Whites [25,26], poverty may lead to financial inequality across racial and ethnic groups [27]. When looking at racial and ethnic differences in economic well-being, African Americans are less likely to be hired [28] and often have disadvantages in income, earnings and asset accumulation compared to Whites [22]. As such, net worth and poverty influence life in a variety of ways. Little is known, however, about the relationship between maternal economic well-being and children’s mental health, particularly with racial/ethnic disparities taken into consideration.

### 1.2. Mental Health in Young Adulthood and Relationships with Mothers

Addressing mental health is important to maintaining a healthy life, improving productivity, and achieving happiness [29]. Many people of all ages experience mental health problems [30]. However, Kessler and Bromet reported that young adults are more likely to report depression than older populations [31]. Moreover, attachment theory states that children develop a close relationship with their mothers over a long-term period. The theory explains children’s need to develop a close relationship with their primary caregiver, such as the mothers in this study, and that maternal care and behaviors greatly influence children’s development [32]. This, in turn, suggests that the maternal factors that influence children’s mental health may be important when those children reach adulthood. Such maternal influences remain important into early adulthood in a variety of ways [33]. Young adults still need care from their mothers [34] and mothers continue to worry about their children even after they leave home for college or work [35]. As such, the relationship with one’s mother may continue to affect young adults’ mental health even as they enter into adulthood.

### 1.3. Economic Well-Being and Mental Health

Assets allow an individual to have economic security and to develop a positive attitude toward their daily life [36], whereas being in debt is negatively associated with depression [37] and overall mental health [38]. Therefore, increasing one’s assets may be beneficial for avoiding mental health problems [39]. For example, individuals who feel that they have sufficient economic resources tend to experience less depression than those who do not [40].

Moreover, poverty has been shown to be associated with depression [12], and individuals in poverty are at increased risk for depressive symptoms [41]. Beyond the individual, the effects of poverty are extensive because all family members experience household financial challenges [42]. Thus, household poverty cannot be viewed strictly as an individual problem because it may influence the mental health of all household members. Given that young adults often still have a close relationship with their mothers and are likely to be influenced by their mothers’ economic status, economic factors such as maternal poverty and net worth may be related to a young adults’ mental health.

### 1.4. Racial Disparities between Maternal Economic Well-Being and Mental Health

Little is known about racial/ethnic disparities in the relationship between maternal economic well-being and mental health in young adulthood, even though associations have been found between net worth and mental health, and poverty and mental health [12]. Generally, African Americans and Hispanics who suffer from economic hardships are at higher risk for mental health problems [12,13], but one study reported contradictory findings, indicating that depressive symptoms among African Americans were less influenced by financial challenges compared to Whites [10]. Few studies, however, have explored this relationship between mothers and their young adult children. Therefore, the purpose of this study is to explore the effect of maternal economic well-being on their children’s mental health outcomes in young adulthood, and to examine the moderating effect of race and ethnicity on the relationship.

## 2. Methods

### 2.1. Sample

The present study used data from the National Longitudinal Survey of Youth 1979 (NLSY79), collected from 1979 to 2012, and the National Longitudinal Survey of Youth 79 for Children and Young Adults (NLSY79 CY), collected from 1986 to 2012. The U.S. Department of Labor conducted both surveys. The NLSY 79 is a nationwide representative dataset with 12,686 participants. The NLSY79 CY recruited the biological children of the females from the NLSY79, and 11,521 children were interviewed. This study used data from both the NLSY79 and NLSY79 CY, collected in 2012. The two datasets were merged, and children were matched with their mothers based on the identification numbers in the NLSY. If young adult siblings were included in the dataset, they were each matched with their mother. Children who were not interviewed or refused to be interviewed were excluded from this study. A total of 4224 pairs were selected for the final sample. The sample included 1970 White, 1376 African American, and 878 Hispanic young adult children. Of the total sample, men made up 47.4% of participants while women accounted for 52.6%.

### 2.2. Measures

Depression. The Center for Epidemiologic Studies Depression Scale (CES-D) was used to measure levels of depression for the young adult children. This scale consists of eleven items that are rated on a four-point Likert-type scale, with response options ranging from 0 “rarely or none of the time (<1 day)” to 3 “most or all of the time (5–7 days).” One of the items, “I was happy,” was reversed prior to using the scale for analysis. Total scores were computed as the sum of all items, with higher scores indicating more depression (Mean = 5.10; SD = 5.03). The CES-D is highly correlated with other measurements for depression [43,44].

Net worth. Respondents reported their assets and debts, and the net worth amount was computed by summing all asset values and subtracting all debts. The NLSY 79 provides the net worth, with imputed missing assets and debt values, and the top 2 percent of all values are top-coded. Respondents were asked to report whether they had an asset or debt and then asked for the value with 15 mid-level groups of questions. The categories are as follows: home value; mortgages; other residential debt; value and debt of farm, business and real estate; market value and debt of vehicles; value of stocks, bonds and mutual funds; value of CDs; value of trusts; value of IRAs; value of 401 k and 403 bs; value of cash savings; value of other assets such as jewelry and collections; and value of all other debts such as credit cards and student loans (Mean = 24.00; SD = 53.95).

Poverty. The poverty variable indicated whether an individual’s income in 2012 was above or below the poverty level. Responses were coded into two groups: those who were above the poverty level (coded = 0) and those who were below the poverty level (coded = 1).

Control variables. Demographic variables included as covariates in this study were age, gender, education, and marital status.

### 2.3. Analysis Strategies

**Figure 1:**
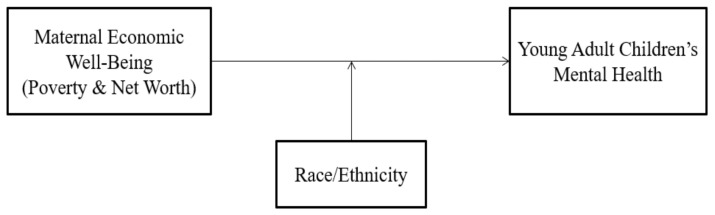
Research Framework.

Analysis of variance (ANOVA) was used to examine racial or ethnic disparities in the baseline variables. Ordinary linear regression and logistic regression analyses were conducted to explore whether there were racial or ethnic disparities in maternal economic well-being after controlling for individual characteristics (research question 1). Ordinary linear regression analyses were used to investigate whether maternal economic well-being was related to their young adult children’s mental health and if this association differed by race/ethnicity (research question 2 and 3). Figure 1 shows the research design, indicating the moderating effect of race and ethnicity on the relationship between maternal economic well-being and children’s mental health outcomes in adulthood. This study employed Statistical Package for the Social Sciences (SPSS) version 22.0 (IBM, Armonk, NY, USA) to examine the research questions.

## 3. Results

Table 1 shows racial and ethnic differences in demographics, maternal economic well-being, and young adult children’s depression. African American young adult children reported higher levels of depression than non-Hispanic Whites and Hispanics. The measures of maternal economic well-being also revealed racial and ethnic disparities in poverty and net worth. Non-Hispanic Whites were less likely to live in poverty than African Americans and Hispanics. African Americans were the group most vulnerable to poverty. The same results were found regarding net worth. Non-Hispanic Whites indicated greater net worth compared to African Americans and Hispanics, and African Americans reported the lowest levels of net worth compared to non-Hispanic Whites and Hispanics. Additionally, both non-Hispanic White mothers and their young adult children were more likely to receive a higher education than African American and Hispanic mother–child dyads. African American mothers and young adult children also reported lower rates of marriage than non-Hispanic White and Hispanic mother–child dyads.

As shown in Table 2, there were significant racial and ethnic disparities in maternal economic well-being. Both African Americans and Hispanics were more likely to have a low net worth (β = −20.16, *p* < 0.001; β = −11.89, *p* < 0.001) and they were more vulnerable to poverty (β = 1.07, OR = 2.94, *p* < 0.001; β = 1.15, OR = 3.17, *p* < 0.001) than non-Hispanic Whites. Higher education was also significantly associated with maternal economic well-being. Individuals with higher education had greater net worth (β = 20.79, *p* < 0.001) and were less likely to be in poverty (β = −1.79, OR = 0.18, *p* < 0.001) than those who did not receive a higher education. Marriage was also significantly associated with economic well-being. Marriage positively affected net worth (β = 19.53, *p* < 0.001) and was associated with a decreased risk of poverty, compared to not being married (β = −1.72, OR = 0.18, *p* < 0.001).

**Figure 2:**
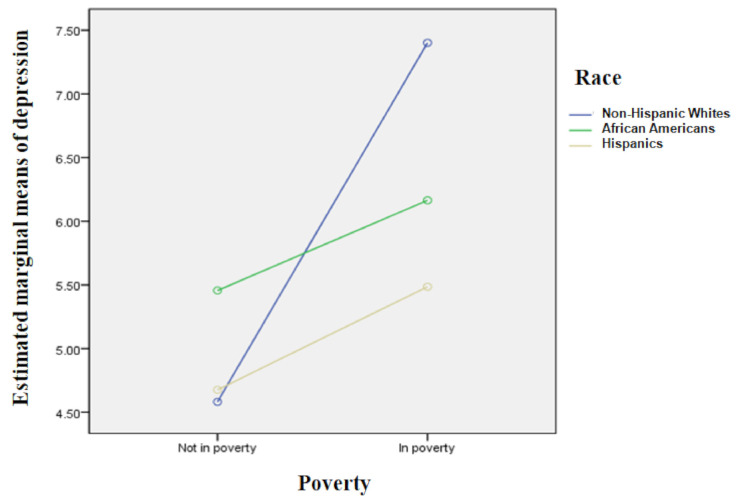
Effects of Maternal Poverty and Race/Ethnicity on Young Adult Children’s Depression.

Table 3 presents racial and ethnic disparities in young adult children’s depression. Model 1 showed that African American young adult children were at greater risk for depression than non-Hispanic Whites (β = 0.84, *p* < 0.001). As demographics were entered into model 2, however, the disparities did not remain significant between African Americans and non-Hispanic Whites. Being female and of an older age were significantly related to depression (β = 0.89, *p* < 0.001; β = 0.11, *p* < 0.001), while individuals with higher education and who were married were less likely to be depressed (β = −1.65, *p* < 0.001; β = –1.81, *p* < 0.001). Further, model 3 indicated that Hispanics had lower levels of depression than non-Hispanic Whites (β = −0.39, *p* < 0.10). Other demographic factors significantly persisted across the young adult children even after controlling for the mother’s demographics. Mothers’ marital status was negatively related to their young adult children’s depression (β = −0.67, *p* < 0.001). Model 4 showed that the racial and ethnic disparities between Hispanics and non-Hispanic Whites for depression remained significant after controlling for maternal economic well-being (β = −0.52, *p* < 0.05). Lower net worth and poverty among mothers were also positively associated with their children’s depression in young adulthood (β = −3.82, *p* < 0.05; β = 0.80, *p* < 0.01), and young adult children’s demographics remained significant. Interaction effects were also found in model 5, indicating that race and ethnicity moderated the relationship between maternal poverty and young adult children’s depression. As shown in Figure 2, depression among non-Hispanic White young adult children was more greatly influenced by maternal poverty than among African American and Hispanic young adult children. Depression among non-Hispanic Whites was highest when their mothers were in poverty (7.40), and at the lowest when their mothers were not in poverty (4.58). In other words, depression among African American and Hispanic young adult children was less influenced by maternal poverty than it was for non-Hispanic Whites.

## 4. Discussion and Conclusions

This study found racial and ethnic disparities in the relationship between maternal economic well-being and young adults’ mental health in a nationwide sample. In examining maternal economic well-being, both African American and Hispanic young adults tended to have lower net worth and tended to be at greater risk of poverty compared to non-Hispanic White young adults. When considering young adults’ mental health, African Americans were more likely to be depressed than non-Hispanic Whites, and identifying as Hispanic was significantly associated with depression after controlling for maternal economic well-being. These findings indicated that mothers’ net worth and poverty status influenced their children’s depression in young adulthood. Interestingly, interaction effects were also found: being African American or Hispanic moderated the relationship between maternal poverty and young adult children’s depression.

The findings about racial and ethnic disparities in maternal economic well-being in this study are consistent with other studies, indicating that African Americans and Hispanics are likely to have lower levels of economic well-being compared to their counterparts [25,45]. While there is a great deal of previous research showing racial and ethnic differences in economic factors [22,27,28], little attention has been paid to women’s economic well-being. Thus, these findings contribute to research regarding women’s economic well-being, accounting for racial and ethnic differences. However, this study specifically addresses net worth rather than income. The difference between assets and income is their time frame [22,46,47]. Assets ensure security, whereas income is used for immediate consumption, thereby making assets a particularly important economic resource for measurement [36]. Unlike previous studies that have focused mainly on income as an indicator for economic well-being [48,49], this study might provide a more specific indicator to measure economic well-being and further evidence about racial and ethnic differences in economic well-being because net worth includes an accumulated amount of income.

Consistent with prior studies reporting that poor economic well-being—such as poverty, low net worth, and lack of income—may be associated with lower mental health [12,37], the current study also reveals the relationship between economic well-being and depression. The findings from this study provide further evidence about the relationship between maternal economic well-being and their young adults’ mental health. While prior research shows the relationship between economic well-being and mental health [10,50,51], little is known about the role of maternal economic well-being. As women’s economic and workforce participation have increased and their contributions to household economic status have become increasingly important in society [5,21], it is necessary to pay more attention to women’s economic well-being as well as its relationship with their children’s mental health in young adulthood. Given that women’s labor force rate is increasing [5], mothers’ influence on their young adult children’s mental health might increase upon consideration of the close relationship between mother and child, based on attachment theory [32]. According to this theory, children with a secure attachment have developed a very close cohesion with their mothers over time and have been exposed to their mother’s care and influence [32]. Therefore, even as the children become young adults, taking maternal economic well-being into consideration is important in understanding their mental health. As shown in this study, young adults whose mothers were in poverty and had lower levels of net worth were more likely to be depressed. Given that household poverty affects all household members [42] and wealth also influences family members’ quality of life, parents’ economic well-being should be understood to explain young adult children’s mental health problems. Particularly, as women’s labor force rate is increasing over time [5], mothers’ economic well-being should be given more attention to understand young adult children’s mental health.

Findings also indicate that race and ethnicity moderate the relationship between maternal economic well-being and young adults’ depression. Although all young adult children, regardless of race or ethnicity, reported a higher risk of depression if their mother experienced poverty, non-Hispanic White young adult children showed a much higher gap in depression levels between their mothers’ being in poverty and not being in poverty, as compared to African American and Hispanic children. In other words, non-Hispanic White children’s mental health may be more likely to be influenced by maternal economic well-being than their counterparts. This might align with evidence of lower socioeconomic status Whites incurring detrimental outcomes from racist policies largely directed toward African Americans [52]. In other words, non-Hispanic White young adult children whose mothers are in poverty might not receive adequate benefits or support, leading them to experience higher levels of depression. Given that there are few studies investigating racial and ethnic disparities in the relationship between maternal economic well-being and young adult children’s mental health, this finding contributes to developing anti-poverty policies targeted at women in order to reduce mental health problems among non-Hispanic White young adult children. That is, providing more opportunities for non-Hispanic White women to engage in the labor market might be beneficial to improving their children’s mental health even when they enter young adulthood. However, as African American young adult children showed higher levels of depression when their mothers were not in poverty compared to non-Hispanic Whites, it is also important to focus on reducing mental health problems among African American young adults.

In sum, this study contributes to a better understanding of the impact of mothers’ economic resources on their young adult children’s mental health. Previous studies have mainly addressed the relationship between fathers and children. However, a new perspective is necessary to more deeply understand young adults’ mental health. In addition, few studies have accounted for parents’ economic influences to explain mental health. The current study fills these research gaps by considering mothers’ influence separate from fathers’ influence. Further, as race and ethnicity moderate the relationship between maternal economic well-being and young adult children’s depression, racial and ethnic disparities should be considered to explain this intergenerational relationship.

## 5. Limitations

Although the current study fills the research gap as discussed above, the findings should be understood in the context of some limitations. First, self-reported net worth might not be an accurate measure of the true value of financial well-being because of self-report bias. Thus, we recommend that future studies use an alternative measure to capture financial well-being more accurately. Second, marital status could not include couples who are not married but have a child. Thus, it is necessary to include all types of households with a child. Third, as a limitation of the use of secondary data, Asian Americans were not included as a racial/ethnic group in this study. Further, more covariates about life circumstances are needed in future studies. Fourth, this study employed one wave of data based on a cross-sectional approach. Thus, it is limited in its ability to explain causal relationships. We suggest that a longitudinal analysis is necessary to more deeply understand the intergenerational relationship examined in this study.

## Figures and Tables

**Table 1:** Descriptive Statistics for Variables Included in the Study.

Variable	African American (*n* = 1376)	Hispanic (*n* = 878)	Non-Hispanic White (*n* = 1970)	Total	*p*
	% or Mean (SD)	% or Mean (SD)	% or Mean (SD)	(*n* = 4224)	
**Young adult child**					
Depression	5.74 (5.05)	4.83 (5.02)	4.78 (4.98)	5.10 (5.03)	a,b
Gender (Female)	52.6%	50.7%	49.9%	50.9%	ns
Age	26.50 (6.30)	25.46 (5.90)	23.93 (5.43)	25.08 (5.93)	a,b,c
Higher education	13.2%	14.4%	20.4%	16.8%	b,c
Marriage	11.3%	16.7%	17.1%	15.1%	a,b
**Mother**					
Poverty	34.7%	30.2%	8.5%	21.6%	a,b,c
Net worth	7.41 (27.08)	17.83 (44.60)	38.38 (66.59)	24.00 (53.95)	a,b,c
Age	50.60 (2.24)	50.60 (2.31)	50.57 (2.19)	50.59 (2.23)	ns
Higher education	26.2%	21.7%	44.0%	33.4%	a,b,c
Marriage	34.6%	49.4%	69.6%	53.9%	a,b,c

Note. a = Significant difference between African American and Hispanic at 0.05; b = Significant difference between African American and non-Hispanic White at 0.05; c = Significant difference between Hispanic and non-Hispanic White at 0.05; Note. The real values of net worth should be multiplied by 10,000.

**Table 2:** Regression Results of Unstandardized Coefficients (standard error) and [Standardized Coefficients] Predicting Net Worth, Logistic Regression Coefficients (Wald) [Expected value] Predicting Poverty.

Variables	Economic Well-Being			
	Net Worth		Poverty	
(Constant)	−99.58 (18.89)		−1.54 (1.09)	
African American	−20.16		1.07	
	(20.10)		(63.70)	
	[−0.17]	***	[2.94]	***
Hispanic	−11.89		1.15	
	(22.31)		(88.36)	
	[−0.09]	***	[3.17]	***
Ages	22.79		0.01	
	(37.11)		(0.23)	
	[0.09]	***	[1.01]	
Higher education	20.79		−1.74	
	(18.15)		(133.98)	
	[0.18]	***	[0.18]	***
Marriage	19.53		−1.72	
	(17.69)		(228.41)	
	[0.18]	***	[0.18]	***

Note. *** *p* < 0.001.

**Table 3:** Regression Results of Unstandardized Coefficients (standard error) and [Standardized Coefficients] Predicting Young Adult Children’s Depression.

Variables	Depression									
	Model 1		Model 2		Model 3		Model 4		Model 5	
(Constant)	4.86 (0.13)		2.35 (0.42)		4.35 (2.03)		3.80 (2.03)		3.77 (2.03)	
**Young adult child**										
African American	0.84		0.29		0.03		−0.14		0.14	
	(0.20)		(0.21)		(0.22)		(0.22)		(0.25)	
	[0.08]	***	[0.03]		[0.00]		[−0.01]		[0.01]	
Hispanic	0.07		−0.21		−0.39		−0.52		−0.30	
	(0.23)		(0.23)		(0.24)		(0.24)		(0.27)	
	[0.01]		[−0.02]		[−0.03]	+	[−0.04]	*	[−0.02]	
Gender (Female)			0.89		0.86		0.87		0.86	
			(0.18)		(0.18)		(0.18)		(0.18)	
			[0.09]	***	[0.09]	***	[0.09]	***	[0.09]	***
Age			0.11		0.10		0.10		0.10	
			(0.02)		(0.02)		(0.02)		(0.02)	
			[0.09]	***	[0.12]	***	[0.11]	***	[0.11]	***
Higher education			−1.65		−1.49		−1.38		−1.39	
			(0.25)		(0.25)		(0.25)		(0.25)	
			[−0.12]	***	[−0.11]	***	[−0.10]	***	[−0.10]	***
Marriage			−1.81		−1.75		−1.74		−1.71	
			(0.28)		(0.28)		(0.28)		(0.28)	
			[−0.13]	***	[−0.12]	***	[−0.12]	***	[−0.12]	***
**Mother**										
Age					−0.02		−0.01		−0.02	
					(0.04)		(0.04)		(0.04)	
					[−0.01]		[−0.01]		[−0.01]	
Higher education					−0.29		−0.10		−0.10	
					(0.20)		(0.20)		(0.20)	
					[−0.03]		[−0.01]		[−0.01]	
Marriage					−0.67		−0.42		−0.41	
					(0.19)		(0.20)		(0.20)	
					[−0.07]	***	[−0.04]	*	[−0.04]	*
Economic well-being										
Net worth							−3.82		−3.47	
							(0.00)		(0.00)	
							[−0.04]	*	[−0.04]	*
Poverty							0.80		20.10	
							(0.24)		(0.47)	
							[0.07]	**	[0.17]	***
Poverty *African American									−1.71	
									(0.56)	
									[−0.11]	**
Poverty *Hispanic									−1.63	
									(0.62)	
									[−0.08]	**

Note. * *p* < 0.05. ** *p* < 0.01. *** *p* < 0.001.

## References

[1] Carlson D.L. (2012). Deviations from desired age at marriage: Mental health differences across marital status. J. Marriage Fam..

[2] Paul K.I., Moser K. (2009). Unemployment impairs mental health: Meta analyses. J. Vocat. Behav..

[3] Rosenfield S., Mouzon D., Aneshensel C.S., Phelan J.C., Bierman A. (2013). Gender and mental health. Handbook of the Sociology of Mental Health.

[4] Cho S.M., Kim E., Lim K.Y., Lee J.W., Shin Y.M. (2015). The effects of maternal depression on child mental health problems based on gender of the child. Community Ment. Health J..

[5] Iversen T., Rosenbluth F. (2005). Gender Socialization: How Bargaining Power Shapes Attitudes (Working Paper no. 2008-0064).

[6] Barrio C., Palinkas L.A., Yamada A.M., Fuentes D., Criado V., Garcia P., Jeste D.V. (2008). Unmet needs for mental health services for Latino older adults: Perspectives from consumers, family members, advocates, and service providers. Community Ment. Health J..

[7] Brennan M., Vega M., Garcia I., Abad A., Friedman M.B. (2005). Meeting the mental health needs of elderly Latinos affected by depression: Implications for outreach and service provision. Care Manag. J..

[8] Mirowsky J., Ross C.E. (1999). Economic hardship across the life course. Am. Sociol. Rev..

[9] Pearlin L.I., Lieberman M.A., Menaghan E.G., Mullan J.T. (1981). The stress process. J. Health Soc. Behav..

[10] Kahn J.R., Pearlin L.I. (2006). Financial strain over the life course and health among older adults. J. Health Soc. Behav..

[11] Saxena S., Thornicroft G., Knapp M., Whiteford H. (2007). Resources for mental health: Scarcity, inequity, and inefficiency. Lancet.

[12] Andersen I., Thielen K., Nygaard E., Diderichsen F. (2009). Social inequality in the prevalence of depressive disorders. J. Epidemiol. Community Health.

[13] Lincoln K.D., Chae D.H. (2010). Stress, marital satisfaction, and psychological distress among African Americans. J. Fam. Issues.

[14] Kalil A., Ziol-Guest K.M., Hawkley L.C., Cacioppo J.T. (2009). Job insecurity and change over time in health among older men and women. J. Gerontol. B Psychol. Sci. Soc. Sci..

[15] Rubery J., Smith M., Fagan C. (1999). Women’s Employment in Europe: Trends and Prospects.

[16] Eastin J., Prakash A. (2013). Economic development and gender equality: Is there a gender Kuznets curve?. World Polit..

[17] Forsythe N., Korzeniewicz R.P., Durrant V. (2000). Gender inequalities and economic growth: A longitudinal evaluation. Econ. Dev. Cult. Chang..

[18] Warner M. (2006). Putting child care in the regional economy: Empirical and conceptual challenges and economic development prospects. Community Dev. J..

[19] Popescu G.H. (2015). The dynamics of social innovation networks. Psychosociological Issues Hum. Resour. Manag..

[20] Popescu G.H. (2016). Gender, work, and wages: Patterns of female participation in the labor market. J. Self-Gov. Manag. Econ..

[21] Ding S., Dong X.Y., Li S. (2009). Women’s employment and family income inequality during China’s economic recession. Fem. Econ..

[22] Oliver M.L., Shapiro T.M. (2006). Black Wealth/White Wealth: A New Perspective on Racial Inequality.

[23] Mossakowski K.N. (2008). Dissecting the influence of race, ethnicity, and socioeconomic status on mental health in young adulthood. Res Aging.

[24] Federal Interagency Forum on Aging-Related Statistics. https://agingstats.gov/docs/PastReports/2012/OA2012.pdf.

[25] Menselson T., Rehkopf D.H., Kubzansky L.D. (2008). Depression among Latinos in the United States: A meta-analytic review. J. Consult. Clin. Psychol..

[26] Toldson I.A., Snitman A. (2010). Editor’s comment: Education parity and economic disparities: Correcting education attainment discrepancies between black people in the United States. J. Negro Educ..

[27] Gilder G. (2012). Wealth and Poverty: A New Edition for the Twenty-First Century.

[28] Western B., Bloome D., Sosnaud B., Tach L. (2012). Economic insecurity and social stratification. Annu. Rev. Sociol..

[29] Ploubidis G.B., Grundy E. (2009). Later-life mental health in Europe: A country-level comparison. J. Gerontol. B Psychol. Sci. Soc. Sci..

[30] McFarland M.J. (2009). Religion and mental health among older adults: Do the effects of religious involvement vary by gender?. J. Gerontol. B Psychol. Sci. Soc. Sci..

[31] Kessler R.C., Bromet E.J. (2013). The epidemiology of depression across cultures. Annu. Rev. Public Health.

[32] Bretherton I. (1992). The origins of attachment theory: John Bowlby and Mary Ainsworth. Dev. Psychol..

[33] Grossmann K., Grossmann K.E., Kindler H., Zimmermann P., Cassidy J., Shaver P.R. (2008). A wider view of attachment and exploration: The influence of mothers and fathers on the development of psychological security from infancy to young adulthood. Handbook of Attachment: Theory, Research, and Clinical Applications.

[34] Belsky J., Cassidy J., Shaver P.R. (1999). Modern evolutionary theory and patterns of attachment. Handbook of Attachment: Theory, Research, and Clinical Applications.

[35] Kobak R., Ferenz-Gillies R., Everhart E., Seabrook L. (1994). Maternal attachment strategies and emotion regulation with adolescent offspring. J. Res. Adolesc..

[36] Zhan M. (2006). Economic mobility of single mothers: The role of assets and human capital development. J. Sociol. Soc. Welf..

[37] Meltzer H., Bebbington P., Brugha T., Jenkins R., McManus S., Stansfeld S. (2010). Job insecurity, socio-economic circumstances and depression. Psychol. Med..

[38] Zurlo K.A., Yoon W., Kim H. (2014). Unsecured consumer debt and mental health outcomes in middle-aged and older Americans. J. Gerontol. B Psychol. Sci. Soc. Sci..

[39] Ssewamala F.M., Han C.K., Neilands T.R. (2009). Asset ownership and health and mental health functioning among AIDS-orphaned adolescents: Findings from a randomized clinical trial in rural Uganda. Soc. Sci. Med..

[40] Wickrama K.A.S., Surjadi F.F., Lorenz F.O., Conger R.D., O’Neal C.W. (2012). Family economic hardship and progression of poor mental health in middle-aged husbands and wives. Fam. Relat..

[41] Lorant V., Croux C., Weich S., Deliege D., Mackenbach J., Ansseau M. (2007). Depression and socio-economic risk factors: 7-year longitudinal population study. Br. J. Psychiatry.

[42] Yoshikawa H., Aber J.L., Beardslee W.R. (2012). The effects of poverty on the mental, emotional, and behavioral health of children and youth: Implications for prevention. Am. Psychol..

[43] Radloff L.S. (1977). The CES-D scale: A self-report depression scale for research in the general population. Appl. Psychol. Meas..

[44] Ross C.E., Mirowsky J. (1989). Explaining the social patterns of depression: Control and problem solving-or support and talking?. J. Health Soc. Behav..

[45] Hughes M., Kiecolt K.J., Keith V.M. (2014). How racial identity moderates the impact of financial stress on mental health among African Americans. Soc. Ment. Health.

[46] Schreiner M. (2004). Measuring Savings (Working Paper No. 04-08).

[47] Wolff E.N. (1995). Top Heavy: A Study of the Increasing Inequality of Wealth in America.

[48] Roberts S., Wyn J., Cahill H. (2015). Ordinary working lives and the “missing midlife” of youth studies. Handbook of Children and Youth Studies.

[49] Patel V. (2007). Mental health in low- and middle-income countries. Br. Med. Bull..

[50] Sun F., Hilgeman M.M., Durkin D.W., Allen R.S., Burgio L.D. (2009). Perceived income adequacy as a predictor of psychological distress in Alzheimer’s caregivers. Psychol. Aging.

[51] Szanton S.L., Thorpe R.J., Whitfield K. (2010). Life course financial strain and health in African-Americans. Soc. Sci. Med..

[52] McGhee H. (2021). The Sum of Us: What Racism Costs Everyone and How We Can Prosper Together.

